# Predicting the naturalistic course in anxiety disorders using clinical and biological markers: a machine learning approach

**DOI:** 10.1017/S0033291720001658

**Published:** 2022-01

**Authors:** Wicher A. Bokma, Paul Zhutovsky, Erik J. Giltay, Robert A. Schoevers, Brenda W.J.H. Penninx, Anton L.J.M. van Balkom, Neeltje M. Batelaan, Guido A. van Wingen

**Affiliations:** 1 Department of Psychiatry, Amsterdam UMC, Vrije Universiteit, Amsterdam Public Health research institute, The Netherlands; 2 GGZ inGeest Specialized Mental Health Care, Amsterdam, The Netherlands; 3 Department of Psychiatry, Amsterdam UMC, Location AMC, University of Amsterdam, Amsterdam Neuroscience, Amsterdam, The Netherlands; 4 Department of Psychiatry, Leiden University Medical Center (LUMC), Leiden, The Netherlands; 5 Department of Psychiatry, University Medical Center Groningen, Groningen, The Netherlands

**Keywords:** agoraphobia, anxiety disorders, classification, generalized anxiety disorder, machine learning, panic disorder, random forest classification, social phobia

## Abstract

**Background:**

Disease trajectories of patients with anxiety disorders are highly diverse and approximately 60% remain chronically ill. The ability to predict disease course in individual patients would enable personalized management of these patients. This study aimed to predict recovery from anxiety disorders within 2 years applying a machine learning approach.

**Methods:**

In total, 887 patients with anxiety disorders (panic disorder, generalized anxiety disorder, agoraphobia, or social phobia) were selected from a naturalistic cohort study. A wide array of baseline predictors (*N* = 569) from five domains (clinical, psychological, sociodemographic, biological, lifestyle) were used to predict recovery from anxiety disorders and recovery from all common mental disorders (CMDs: anxiety disorders, major depressive disorder, dysthymia, or alcohol dependency) at 2-year follow-up using random forest classifiers (RFCs).

**Results:**

At follow-up, 484 patients (54.6%) had recovered from anxiety disorders. RFCs achieved a cross-validated area-under-the-receiving-operator-characteristic-curve (AUC) of 0.67 when using the combination of all predictor domains (sensitivity: 62.0%, specificity 62.8%) for predicting recovery from anxiety disorders. Classification of recovery from CMDs yielded an AUC of 0.70 (sensitivity: 64.6%, specificity: 62.3%) when using all domains. In both cases, the clinical domain alone provided comparable performances. Feature analysis showed that prediction of recovery from anxiety disorders was primarily driven by anxiety features, whereas recovery from CMDs was primarily driven by depression features.

**Conclusions:**

The current study showed moderate performance in predicting recovery from anxiety disorders over a 2-year follow-up for individual patients and indicates that anxiety features are most indicative for anxiety improvement and depression features for improvement in general.

## Introduction

Anxiety disorders are characterized by highly heterogeneous clinical course trajectories. After 2 years, the prognosis varies across disorders with remittance rates of 72.5% for panic disorder without agoraphobia, 69.7% for generalized anxiety disorder, 53.5% for social phobia and 52.7% for panic disorder with agoraphobia (Hendriks, Spijker, Licht, Beekman, & Penninx, 2013). Remitted patients experience a relatively benign course with moderate remaining symptom severity, disability and a low subjective need for care (Batelaan, Rhebergen, Spinhoven, van Balkom, & Penninx, 2014; Spinhoven et al., 2016; van Beljouw, Verhaak, Cuijpers, van Marwijk, & Penninx, 2010). However, around 60% of patients have persistent symptoms, relapses, or chronic disease up to 6 years after the diagnosis (Batelaan et al., 2014; Spinhoven et al., 2016). Disease course in these patients is often characterized by substantial levels of disability. Predicting long-term disease course can be seen as an important step towards personalized medicine (Steyerberg, 2009). This would make targeted treatment efforts viable, in which treatments are tailored towards the individual risk for a poor disease outcome (McGorry, Ratheesh, & O'Donoghue, 2018). However, in anxiety disorders, there is a lack of robust course predictors. For instance, different DSM anxiety disorder diagnoses were shown to be poorly predictive of subsequent course (Batelaan et al., 2014). In current clinical practice, in the absence of valid risk prediction models, course prediction relies solely on clinician's opinions, which show poor accuracy (Randall, Sareen, Chateau, & Bolton, 2019).

Several clinical, psychological, biological, sociodemographic and lifestyle markers are related to the disease course. For instance, higher baseline severity of anxiety symptoms, presence of somatic or psychiatric comorbidity, and higher levels of disability are linked to worse outcomes at 1-year (van Beljouw et al., 2010), 2-year (Batelaan et al., 2014; Hendriks et al., 2013; Scholten et al., 2013), 6-year (Spinhoven et al., 2016), and 12-year follow-up (Bruce et al., 2005). Contrastingly, some authors suggest the same factors lead to better initial treatment results (Baldwin & Tiwari, 2009; Rodriguez et al., 2006). Also, a chronic duration of anxiety was linked to worse outcomes in most studies (Batelaan et al., 2014; Hendriks et al., 2013; Scholten et al., 2013; Spinhoven et al., 2016), while not showing any effect on disease course in another study (Nay, Brown, & Roberson-Nay, 2013). Most studies showed that a younger age at onset was associated with a chronic course (Batelaan et al., 2014; Beesdo-Baum et al., 2012; Rodriguez et al., 2006), while others showed no such age effect (Nay et al., 2013; Scholten et al., 2013). Inconsistent findings are likely due to methodological differences between studies. Other factors possibly related to worse disease course were duration of untreated illness (Baldwin & Tiwari, 2009), the use of anti-anxiety medication (Bruce et al., 2005; Scholten et al., 2013), and presence of childhood trauma (Asselmann & Beesdo-Baum, 2015; Batelaan et al., 2014; Scholten et al., 2013). Psychological factors that negatively impact anxiety disorder disease course up till 6-year follow-up included high neuroticism (Asselmann & Beesdo-Baum, 2015; Scholten et al., 2013; Spinhoven et al., 2016), low extraversion (Spinhoven et al., 2016), high anxiety sensitivity (Asselmann & Beesdo-Baum, 2015; Scholten et al., 2013), high levels of worrying (Spinhoven et al., 2016), and low mastery (Asselmann & Beesdo-Baum, 2015; Scholten et al., 2013). Only a few studies linked biological parameters to disease course in anxiety disorders: C-reactive protein (CRP) levels were longitudinally associated with anxiety symptoms (Copeland, Shanahan, Worthman, Angold, & Costello, 2012)**,** increasing cortisol levels were linked to higher 6-month anxiety severity in girls (Schiefelbein & Susman, 2006), and lower Brain-Derived Neurotropic Factor (BDNF) levels were found in patients with a poor response to treatment (Kobayashi et al., 2005). However, most research into biological parameters for anxiety disorders was done cross-sectionally, showing that anxiety disorder status is linked to higher CRP-levels (Copeland et al., 2012; Pitsavos et al., 2006; Vogelzangs, Beekman, De Jonge, & Penninx, 2013), higher metabolic syndrome markers (Carroll et al., 2009; Kahl et al., 2015; Perez-Cornago, Ramírez, Zulet, & Martinez, 2014), higher tumour necrosis factor-*α* (TNF-*α*) levels (Hoge et al., 2009; Pitsavos et al., 2006), and lower BDNF levels (Molendijk et al., 2012). Inconsistently, anxiety symptoms were linked to both higher (Zoccola, Dickerson, & Yim, 2011) and lower (O ’Donovan et al., 2010) cortisol, as well as higher (Hoge *et al*. 2009; O ’Donovan *et al*. 2010; Pitsavos *et al*. 2006) and lower (Vogelzangs *et al*. 2013) interleukin-6 (IL-6) measurements. Finally, sociodemographic and lifestyle factors such as education years (van Beljouw et al., 2010), age (Asselmann & Beesdo-Baum, 2015; Catarino et al., 2018), partner status (Asselmann & Beesdo-Baum, 2015; Batelaan et al., 2014), social support (van Beljouw et al., 2010), smoking status (Bruce et al., 2005), nicotine dependency (Nay et al., 2013), current financial problems (Nay et al., 2013), employment status (van Beljouw et al., 2010), and income (van Beljouw et al., 2010) were associated with anxiety disorder disease course. In spite of these many variables that predict disease course at the group level, it is not known whether this translates to accurate predictions for individual patients. Currently, no encompassing model exists with sufficient sensitivity and specificity in disease course prediction to be feasible for use at the level of the individual patient.

A possible explanation for the lack of accuracy in course prediction in anxiety disorders is the complex, multicausal aetiology of anxiety disorders. Univariable and multivariable analyses of predictors of disease course showed low levels of explained variance (Bokma, Batelaan, Hoogendoorn, Penninx, & van Balkom, 2020). Furthermore, the inference is typically done on the group-level which does not allow for generalizable statements for the single individual. Multivariable machine learning (ML) methods provide a possible solution for this problem, as they are well-suited for solving problems with high numbers of predictors in complex, multicausal disorders (Iniesta, Stahl, & McGuffin, 2016). The use of ML in the field of psychiatry may have great potential for its application in the prediction of disease course trajectories (Hahn, Nierenberg, & Whitfield-Gabrieli, 2017). Prediction of the disease course can be regarded as a ‘classification’ problem, which can be solved using supervised algorithms (Deo, 2015). In these, algorithms are trained on patients with known predictor and outcome variables to derive a function that can be applied to unseen patients to predict their outcome based on the values of their predictor variables. In anxiety disorders, supervised algorithms were applied a few times cross-sectionally, to relate predictors from various domains to current disease status (Woo, Chang, Lindquist, & Wager, 2017) or to predict short-term treatment effects (Lueken & Hahn, 2016). To our best knowledge, however, no studies applied supervised ML algorithms to predict the disease course in anxiety disorders.

The aim of this study was to predict long-term anxiety disorder course, using an ML approach applied to clinical, psychological, biological, sociodemographic and lifestyle baseline data. Specifically, we investigated the utility of a random forest classifier (RFC) (Breiman, 2001) to predict clinical course in patients with any baseline anxiety disorder. Our main outcome was recovery from anxiety disorders at 2-year follow-up. As secondary outcome recovery from all common mental disorders (CMDs) at 2-year follow-up was used. CMDs include anxiety disorders, but also depressive disorders and substance use disorders as these disorders often co-occur, show diagnostic instability over time (Hovenkamp-Hermelink et al., 2016; Lamers et al., 2011; Scholten et al., 2016; Verduijn et al., 2017), and recovery from one but not the other does not index a major improvement in health. Finally, we assessed which predictor domains contributed most to disease course predictions. We hypothesized that RFCs using a wide array of baseline data from different domains would yield adequate 2-year recovery predictions for both outcomes. Furthermore, we hypothesized that the combination of the five domains would yield the best predictions.

## Methods

### Study sample

The participants in this study were selected from the multi-site Netherlands Study of Depression and Anxiety (NESDA), an ongoing naturalistic cohort study into the course of depression and anxiety. The baseline sample consists of 2981 participants who were recruited from the community, primary care and specialized mental health care centres. All participants had a lifetime or current depressive disorder or anxiety disorder diagnosis (*n* = 2329, 78.1%) or were healthy controls (*n* = 652, 21.9%). NESDA allowed for the presence of comorbid psychiatric disorders, with the exception of psychotic disorders, obsessive-compulsive disorder, post-traumatic stress disorder, bipolar disorders, or severe substance use disorders. Exclusion criterion consisted of insufficient proficiency of the Dutch language. Baseline data collection was performed in 2004–2007 and was followed by 1-year, 2-year, 4-year, 6-year, and 9-year follow-up measurements. Full descriptions of the design of NESDA were published previously (Penninx et al., 2008). The study protocol was approved by the Ethical Review Board of all participating institutes and written informed consent was obtained from all participants.

For the purpose of this study, patients with current (6-month) panic disorder (PD, with or without agoraphobia), generalized anxiety disorder (GAD) or social anxiety disorder (SAD) diagnoses at baseline were selected (*n* = 1206). In our sample, psychiatric comorbidity was allowed. The diagnosis was established according to DSM-IV criteria with the Composite International Diagnostic Interview (CIDI, version 2.1) (American Psychiatric Association, 2000; Wittchen, 1994; World Health Organization, 1998). From these patients, 212 were excluded due to missing diagnostic information at 2-years follow-up. A further 107 patients were removed due to having more than 20% missing variables across predictor variables at baseline. This yielded a final sample of 887 anxiety disorder patients with sufficient data available. Excluded patients showed comparable symptom severity at baseline – mean anxiety severity (Beck's Anxiety Inventory; BAI): 20.35 ± 11.74 *v.* 18.30 ± 10.48, *t* = 1.81, *p* = 0.07; mean depression severity (Inventory of Depressive Symptomatology-Self Report; IDS-SR): 30.71 ± 12.65 *v.* 29.39 ± 12.65, *t* = 0.97, *p* = 0.33. Excluded patients were younger (mean age: 38.25 ± 12.05 *v.* 41.92 ± 12.20 years, *t* = 4.62, *p* < 0.001), and had a lower mean number of education years: 11.03 ± 3.15 *v.* 11.88 ± 3.35, *t* = 3.97, *p* < 0.001, consistent with differences across the whole NESDA sample (Lamers et al., 2012). Gender did not differ between excluded and included patients (% female in excluded sample 68.2%, in included sample 66.8%, χ^2^ = 0.22, *p* = 0.64).

### Investigated classifications

Two distinct classification tasks predicting outcomes at 2-year follow-up were performed. Both were binary classification tasks predicting (1) recovery from anxiety disorders or (2) recovery from all CMDs. Anxiety disorders were defined as either PD, agoraphobia, GAD, or SAD. Recovery from anxiety disorders was deemed present if no anxiety disorder diagnoses persisted at follow-up. These diagnoses referred to all follow-up anxiety disorders, not only the index disorder(s). Anxiety disorders, dysthymia, major depressive disorder (MDD) and alcohol dependency are sometimes collectively referred to as CMDs (Ormel et al., 2013; Vollebergh et al., 2001). For the purpose of this study, we defined recovery from all CMDs if at follow-up no anxiety disorders, MDD, dysthymia or alcohol dependency diagnoses were present. Assessment of CMDs is relevant as it is evident from population-based studies that depressive disorders and alcohol dependency are the most commonly occurring comorbidities in anxiety disorders (Alonso & Lépine, 2007; Judd et al., 1998; Wittchen, Kessler, Pfister, & Lieb, 2000), rates of diagnostic instability across anxiety disorders, depressive disorders and alcohol dependency are high (Gustavson et al., 2018; Hovenkamp-Hermelink et al., 2016; Scholten et al., 2016) and recovery from one but not the other does not imply a major improvement in health. We assessed recovery from anxiety disorders as a primary outcome measure and recovery from all CMDs as a secondary outcome measure. These two outcome measures describe recovery in a narrow and a broad perspective (Verduijn et al., 2017).

### Baseline predictor variables

At baseline, a wide array of putative predictors from five domains (clinical, psychological, sociodemographic, biological and lifestyle) were selected, yielding a total of 651 variables. In our analyses, only information at the individual item level was used. Total summary scores for questionnaires were not calculated, as these would be correlated to the individual items. The exception was the NEO Five-Factor Inventory (NEO-FFI), as its domains (e.g. neuroticism) are of specific clinical relevance. Items were excluded if more than 20% of patients were missing the corresponding item. This resulted in the inclusion of 569 predictors at baseline (see Table 1). If a variable did not apply for a patient, it was re-coded as a new category for ordinal or nominal variables or as 0 for continuous variables (all continuous variables were positive). Such an encoding allowed to maintain the variable for classification and encoded it with a not naturally occurring value implying that this variable did not apply for this patient. All additional missing variables were imputed using median/mode imputation calculated on the training set (see below) to obtain a full data set. No variable had more than 10% missing values before imputation was applied. Additional information about measurement instruments, variable scoring and collection can be found in the Supplementary Methods. We investigated the predictive capability of all domains individually and the combination of all five domains.

**Table 1.** Included baseline predictor variables across the five predictor domains

Domain	Timespan	Constructs (no of items)	Measurement instruments
Clinical domain (311 variables)	Current	Common mental disorder diagnoses (25), pathological worrying (11), phobic concerns (15), disability (35), all psychotropic medication, by classes (13).	WHO-Composite International Diagnostic Interview (CIDI), Penn State Worry Questionnaire (PSWQ), Fear Questionnaire (FQ), WHO-Disability Assessment Schedule II (WHO-DAS), according to Anatomical Therapeutic Chemical (ATC) codes.
	Past week	Depressive symptoms (28), general distress and somatization (32), mood and anxiety symptoms (30), suicidal ideation (5).	Inventory of Depressive Symptomatology-SR (IDS-SR), Four-Dimensional Symptom Questionnaire (4DSQ), Mood and Anxiety Scoring Questionnaire (MASQ), Suicidal Ideation Scale (SSI).
	Past four weeks	Anxiety symptoms (21), sleep quality (6).	Beck Anxiety Inventory (BAI), Insomnia Rating Scale (ISR),
	Past six months	Perceived need for care (14).	Perceived Need for Care Questionnaire.
	Past three years	Previous psychotropic medication, by classes (6).	According to ATC codes.
	Past four years	Anxiety duration, months (1).	Life Chart Interview (LCI).
	Lifetime	Anxiety and depressive disorders diagnoses (14), bipolar symptoms (13), number of negative life-events (1), childhood trauma (3), convictions about the importance of care and past experiences with care (36).	CIDI, Mood Disorder Questionnaire (MDQ), Brugha questionnaire, NEMESIS questionnaire, QUality Of care Through the Eyes of the patient (QUOTE): Anxiety/Depression version.
Psychological domain (131 variables)	Current	Anxiety sensitivity (16), cognitive reactivity to sadness (34), mastery (5), personality structure according to the Five Factor (76).	Anxiety Sensitivity Index, Leiden Index of Depression Sensitivity, Pearlin Mastery, NEO Five-Factor Inventory.
Sociodemographic domain (71 variables)	Current	Demographic characteristics (6), employment status (5), marital status (2), sexual preference (1), housing status (5), family and household decomposition (6), income (11), religion (1), leisure activities (20), loneliness (11), social support (3).	Self-report questionnaires, de Jong-Gierveld loneliness scale, Close Person Inventory.
Biological domain (49 variables)	Current	Number of chronic diseases (2), chronic pain (1), menstrual cycle status (4), Body Mass Index (1), hip/waist circumference ratio (2), blood pressure (7), handedness (1), hand-grip strength (2), current fever or cold (2), autonomic nervous system function (6), blood plasma measures, including CRP, TNF-*α*, BDNF, and IL-6 (21).	Chronic graded pain scale, OMRON M4 IntelliSense digital blood pressure monitor, Jamar dynamometer, Vrije Universiteit Ambulatory Measuring System.
Lifestyle domain (7 variables)	Current	Smoking status (1), psychoactive substances use (1), amount of alcohol consumption (1), levels of physical exercise (4).	Fagerström Test for Nicotine Dependence, Alcohol Use Disorders Identification Test, International physical activity questionnaire.

### Machine learning algorithm

RFCs (Breiman, 2001) were used in all analyses. RFCs have been shown to perform well on many different machine learning problems (Fernández-Delgado, Cernadas, Barro, & Amorim, 2014), specifically in biomedical sciences (Olson, Cava, Mustahsan, Varik, & Moore, 2018). An RFC is built as an ensemble of many decision trees (Breiman, Friedman, Olshen, & Stone, 1984) which themselves are trained by considering random subsamples of variables and patients for each tree. Such a procedure leads to improved and robust prediction performance in comparison to individual trees (Breiman, 2001). Details on hyperparameters used in the analysis can be found in the Supplementary Methods. All analyses were implemented using the scikit-learn (version 0.20.2) (Pedregosa et al., 2011) and imbalanced-learn toolboxes (version 0.4.3) (Lemaître, Nogueira, & Aridas, 2017) in the Python programming language (version 3.7.2).

### Evaluation

To evaluate the performance of our classifiers 10-times-repeated-10-fold-cross-validation was applied. In this procedure, the data set is repeatedly (*n* = 100) divided into disjoint training (90% of data) and test (10% of data) sets and the RFC is only fit on the training data and evaluated on the independent test data. The final performance is obtained as an average across all test set evaluations. We measured performance as area-under-the-receiver-operator-curve (AUC). In addition, we calculated sensitivity, specificity, balanced accuracy – average between sensitivity and specificity – and positive/negative predictive values. To further validate our classification performance label-permutation tests (*n* = 1000) of average AUC values were performed (Ojala & Garriga, 2010). The obtained *p* values were Bonferroni-corrected across five individual and one combination of all domains and alpha was set to 0.05.

To systematically compare the performance of different predictor domains patients were distributed in exactly the same way for each of the classifications, i.e. the train and test set of any cross-validation iteration included the same patients for each predictor domain. This allowed the calculation of normalized average differences in AUC scores across cross-validation iterations for each pair of predictor domains (including the combination of all domains). Non-parametric sign-flipping tests (*n* = 10 000) were then employed to derive *p* values which were Bonferroni-corrected for 30 comparisons with alpha set to 0.05.

### Variable importance

In addition to its strong classification performance RFCs allow to quantify the importance of each variable towards the classification task (Breiman, 2001). However, the standard calculation of variable importance has been shown to be biased (Strobl, Boulesteix, Zeileis, & Hothorn, 2007) and a permutation-based variable importance scheme has been suggested instead (Altmann, Toloşi, Sander, & Lengauer, 2010; Hapfelmeier & Ulm, 2013; Strobl et al., 2007). Following this approach, we calculated *p* values for each variable by permuting (*n* = 1000) every variable separately. The computed *p* values were then corrected according to the false discovery rate (FDR) (Benjamini & Hochberg, 2000) and significance was set to 0.05. Given that variable importance was calculated every cross-validation iteration, important variables were defined as variables which were consistently significant under FDR for at least 50% of all cross-validation iterations. This very stringent procedure for identifying important variables was employed to calculate valid variable importance information specific to the classification task. Variable importance were only investigated for the classifications using the data from the combination of all domains. In addition, we investigated differences in the average rankings of important variables between the two classification tasks. A detailed description of this approach can be found in the Supplementary Methods.

## Results

At 2-year follow-up, 484 patients (54.6%) recovered from anxiety disorders, and 362 patients (40.8%) did not have any CMD. Baseline clinical, psychological, sociodemographic, biological and lifestyle variables are provided for patients with and without anxiety disorders at follow-up (Table 2) and for patients with and without CMD at follow-up (online Supplementary Table 1). Various clinical and psychological variables showed differences between the two groups. By contrast, biological and lifestyle status did not differ between the two groups.

**Table 2.** Baseline characteristics of anxiety disorder sample, group comparisons between patients who had no anxiety disorder (*n* = 484) at 2-year follow-up and patients who did (*n* = 403)

Baseline characteristics	Recovered at 2-year follow-up (*n* = 484)	Persistent disorders at 2-year follow-up (*n* = 403)	Statistics	*p*
*Clinical domain*				
PD diagnosis	176 (36.4%)	192 (47.6%)	χ^2^ = 11.52	**<0**.**001**
Agoraphobia diagnosis	141 (29.1%)	176 (43.7%)	χ^2^ = 20.24	**<0**.**001**
SAD diagnosis	196 (40.5%)	212 (53.6%)	χ^2^ = 12.98	**<0**.**001**
GAD diagnosis	141 (29.1%)	136 (33.7%)	χ^2^ = 2.18	0.14
MDD diagnosis	174 (36.0%)	188 (46.7%)	χ^2^ = 10.42	**0**.**001**
Dysthymia diagnosis	58 (12.0%)	88 (21.8%)	χ^2^ = 15.53	**<0**.**001**
Use of psychotropic medication, *current*	345 (71.3%)	294 (73.0%)	χ^2^ = 0.31	0.58
Avoidance behaviour severity, *mean FQ*, *current*	31.76 ± 18.21	40.90 ± 20.07	*t* = −6.98	**<0**.**001**
Pathological worrying severity, *mean PSWQ*, *current*	35.95 ± 9.91	39.56 ± 9.39	*t* = −5.52	**<0**.**001**
Suicidal thoughts, *SSI*, *past week*	72 (14.9%)	111 (27.5%)	χ^2^ = 21.55	**<0**.**001**
Level of distress, *mean 4DSQ*, *past week*	16.03 ± 8.94	19.85 ± 8.75	*t* = −6.39	**<0**.**001**
Depressive symptoms severity, *mean IDS-SR*, *past week*	26.58 ± 12.00	32.78 ± 12.59	*t* = −7.48	**<0**.**001**
Sleep disturbances, *mean ISR, past four weeks*	9.39 ± 5.15	10.19 ± 5.24	*t* = −2.28	**0**.**02**
Anxiety symptoms severity, *mean BAI*, *past month*	15.97 ± 9.36	21.10 ± 11.05	*t* = −7.49	**<0**.**001**
Percentage of time spent with anxiety symptoms, *LCI, past 4 years*	43.81% ± 33.20	54.04% ± 34.20	*t* = −4.37	**<0**.**001**
History of childhood life events^a^	89 (18.4%)	74 (18.4%)	χ^2^ = 0.00	0.99
History of childhood trauma^b^	258 (53.3%)	247 (61.4%)	χ^2^ = 5.93	**0**.**02**
History of serious suicide attempts	66 (13.7%)	87 (21.6%)	χ^2^ = 9.57	**0**.**002**
*Psychological domain*				
Neuroticism, *mean NEO-FF subscale*	40.46 ± 6.89	43.66 ± 6.73	*t* = −6,95	**<0**.**001**
Extraversion, *mean NEO-FFI subscale*	34.70 ± 6.51	32.43 ± 6.82	*t* = 5.06	**<0**.**001**
Conscientiousness, *mean NEO-FFI subscale*	40.88 ± 6.45	39.23 ± 6.36	*t* = 3.82	**<0**.**001**
Agreeableness, *mean NEO-FFI subscale*	43.38 ± 5.37	42.59 ± 5.27	*t* = 2.20	**0**.**03**
Openness, *mean NEO-FFI subscale*	38.25 ± 6.03	38.04 ± 6.32	*t* = 0.51	0.61
Cognitive reactivity to sadness, *mean LEIDS*	40.80 ± 17.98	46.76 ± 17.98	*t* = −4.91	**<0**.**001**
Anxiety sensitivity, *mean ASI*	33.63 ± 9.47	36.58 ± 10.43	*t* = −4.35	**<0**.**001**
Mastery, *mean Mastery scale*	15.77 ± 4.02	13.89 ± 4.06	*t* = 6.90	**<0**.**001**
*Sociodemographic domain*				
Age in years	41.88 ± 12.09	41.97 ± 12.34	*t* = −0.11	0.91
Education years	12.02 ± 3.29	11.70 ± 3.41	*t* = 1.43	0.15
Female gender	329 (68.0%)	276 (68.5%)	χ^2^ = 0.03	0.87
Currently employed	280 (57.9%)	206 (51.1%)	χ^2^ = 4.03	**0**.**05**
Has children	268 (55.4%)	212 (52.6%)	χ^2^ = 0.68	0.41
Current severe loneliness	47 (9.7%)	58 (14.4%)	χ^2^ = 4.57	**0**.**03**
*Biological domain*				
Number of chronic somatic diseases	0.67 ± 0.89	0.72 ± 0.95	*t* = −0.82	0.41
Chronic pain with high disability	100 (20.7%)	121 (30.0%)	χ^2^ = 10.31	**0**.**001**
BMI	25.46 ± 4.72	25.71 ± 5.52	*t* = −0.74	0.46
Mean heart rate (bpm)	71.70 ± 9.59	72.05 ± 10.12	*t* = −0.52	0.60
Systolic blood pressure (mmHg)	136.3 ± 20.63	135.9 ± 17.97	*t* = 0.31	0.76
CRP (mg/L, *n* = 876)	2.67 ± 4.05	3.12 ± 6.29	*t* = −1.26	0.21
IL-6 (pg/ml, *n* = 876)	1.28 ± 3.00	1.43 ± 3.15	*t* = −0.69	0.49
TNF-*α* (pg/ml, *n* = 871)	1.07 ± 1.28	1.04 ± 1.12	*t* = 0.41	0.69
BDNF(ng/ml, *n* = 865)	9.18 ± 3.64	9.20 ± 3.46	*t* = −0.08	0.94
*Lifestyle domain*				
Former smoker	153 (31.6%)	119 (29.5%)	χ^2^ = 2.86	0.24
Current smoker	174 (36.0%)	167 (41.4%)		
Low physical activity, *past week*	103 (22.7%)	98 (25.3%)	χ^2^ = 1.84	0.40
High physical activity, *past week*	156 (34.4%)	117 (30.2%)		
Any substance use, *past week*	33 (6.8%)	33 (8.2%)	χ^2^ = 0.60	0.44
Hazardous drinking or alcohol dependency,^c^ *past year*	109 (22.6%)	87 (21.6%)	χ^2^ = 0.13	0.71

PD, panic disorder; SAD, social anxiety disorder; GAD, generalized anxiety disorder; MDD, major depressive disorder; FQ, Fear Questionnaire; PSWQ, Penn State Worry Questionnaire; SSI, Suicidal Ideation Scale; 4DSQ, Four-Dimensional Symptom Questionnaire; IDS-SR, Inventory of Depressive Symptomatology-SR; ISR, Insomnia Rating Scale; BAI, Beck's Anxiety Inventory; LCI, life chart interview; NEO-FFI, NEO Five-Factor Inventory; LEIDS, Leiden Index of Depression Sensitivity; ASI, Anxiety Sensitivity Index; BMI, Body Mass Index; CRP, c-reactive protein; IL-6, interleukin-6; TNF-*α*, tumour necrosis factor-*α*; BDNF, Brain-Derived Neurotrophic Factor. *p* values shown in bold are <0.05.

a Childhood life events (<16 years of age) were parental divorce, being placed in a juvenile prison, raised in a foster family, placed in a child home, death of a parent.

b Childhood trauma included emotional neglect, psychological abuse, physical abuse, and sexual abuse.

c As measured with the AUDIT. Scores above 8 are reflective of hazardous drinking, scores at 13 or higher (females) and 15 or higher (males) are indicative of probable alcohol dependency.

### Recovery from anxiety disorders

#### Classification performance

**Fig. 1.**
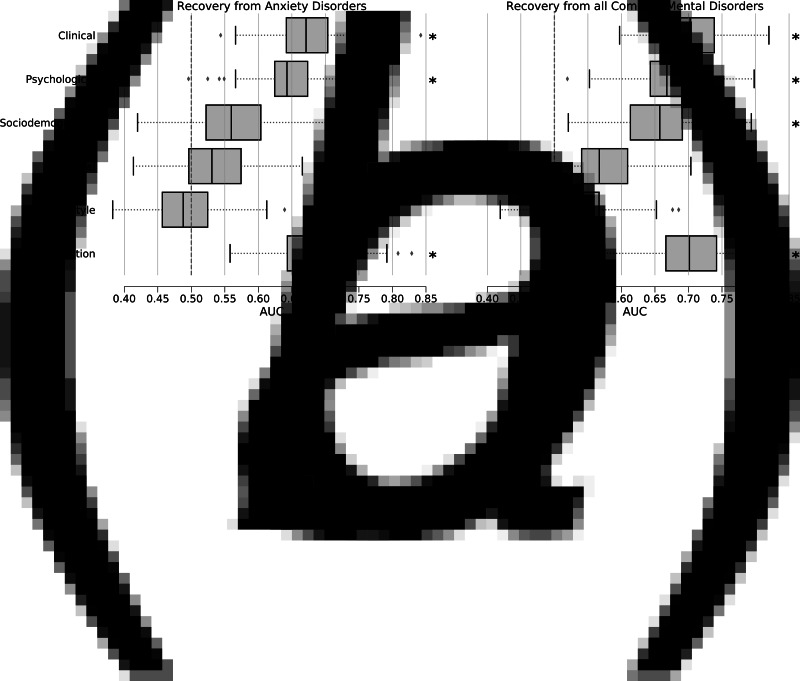
Classification performance of random forest classifiers. Performance is quantified by area-under-the-receiver-operator-curve (AUC) values calculated for each test set of all cross-validation iterations and is shown in box-and-whisker plots for all data domains. (a) Performance of the recovery from anxiety disorders prediction,(b) Performance of the recovery from all common mental disorders prediction. Asterisks mark a significant classification performance according to label-permutation tests (*n* = 1000) and Bonferroni-correction for six tests.The dashed line indicates chance-level performance.

Results of our evaluation of the RFC when predicting recovery from anxiety disorders are reported in Table 3 and Fig. 1*A*. AUC values for the predictor domains ranged from 0.49 to 0.67 with significant (*p_Bonferroni_* < 0.05) AUC values obtained for the clinical (0.67), and psychological (0.65) domains, as well as for the combination of all domains (0.67). Classification accuracies were small to moderate with the highest accuracy achieved by the combination of all domains (62.4%) with a sensitivity of 62.0% and specificity of 62.8%. In addition, we investigated the performance of the RFC for subgroups of patients who had any comorbidity (MDD, dysthymia, or alcohol dependency, n = 252 recovered, n = 248 persistent) at baseline and for patients who did not ( n = 232 recovered, n = 155 persistent). For that, the RFC trained on all data domains and all patients of the training set was evaluated within the two subgroups on the test set separately. The RFC obtained an average AUC of 0.64 within the no-comorbidity group and an AUC of 0.68 within the comorbidity group showing slightly increased performance for predictions within the comorbidity group.

**Table 3.** Evaluation of the 2-year recovery from anxiety disorders classification [mean (s.d.)]

Domains	AUC	Accuracy	Sensitivity	Specificity	PPV	NPV
Clinical	0.67 (0.05)*	61.7 (4.4)	61.5 (6.3)	61.9 (7.6)	0.66 (0.05)	0.57 (0.04)
Psychological	0.65 (0.05)*	61.0 (4.5)	60.0 (6.4)	61.9 (7.5)	0.66 (0.05)	0.56 (0.05)
Socio-demographic	0.56 (0.06)	53.1 (5.1)	49.7 (7.4)	56.5 (7.5)	0.58 (0.06)	0.48 (0.05)
Biological	0.53 (0.06)	52.7 (4.9)	50.3 (6.8)	55.0 (7.6)	0.57 (0.05)	0.48 (0.05)
Lifestyle	0.49 (0.05)	50.2 (4.3)	46.6 (5.5)	53.7 (7.6)	0.55 (0.05)	0.46 (0.04)
Combination	0.67 (0.05)*	62.4 (4.6)	62.0 (6.1)	62.8 (7.5)	0.67 (0.05)	0.58 (0.05)

AUC, area-under-receiver-operator-curve; PPV, positive predictive value; NPV, negative predictive value; *p_Bonferroni_ < 0.05.

*p* values shown in bold are <0.05.

#### Domain comparisons

When comparing different domains according to their AUC a clear ordering was observed: The clinical domain outperformed every other domain except for the combination of all domains (*p_Bonferroni_* < 0.05), the psychological domain outperformed the sociodemographic, biological, and lifestyle domains (*p_Bonferroni_* < 0.05), the sociodemographic domain outperformed the biological and lifestyle domains (*p_Bonferroni_* < 0.05), and the biological domain outperformed the lifestyle domain (*p_Bonferroni_* < 0.05). The combination of all domains was better than any domain except for the clinical domain (*p_Bonferroni_* < 0.05).

#### Variable importance

Consistently selected significant variables (*N* = 17) identified through a permutation-based variable importance calculation of the RFC are reported in online Supplementary Table 2. Only variables from the clinical and psychological domain were selected. These variables were derived from different measurement instruments (BAI, IDS-SR, Fear Questionnaire (FQ), NEO-FFI, WHO-Disability Assessment (WHO-DAS), Four-Dimensional Symptom Questionnaire (4DSQ), Mastery scale) but all referred to characteristic anxiety symptoms, with an emphasis on anxious arousal items.

### Recovery from all common mental disorders

#### Classification performance

Results of the second classification procedure predicting recovery from CMDs are reported in Table 4 and Fig. 1*B*. AUC values ranged from 0.53 to 0.70 with significant (*p_Bonferroni_* < 0.05) AUC values obtained for the clinical (0.70), psychological (0.67), and sociodemographic domain (0.65) as well as the combination of all domains (0.70). The highest accuracy was achieved by the combination of all domains (63.4%) with a sensitivity of 64.6% and a specificity of 62.3%. As in the case of the prediction of the recovery from anxiety disorders, we investigated the performance of the RFC for subgroups of patients who had (n = 164 recovered, n = 336 persistent) or did not (n = 198 recovered, n = 189 persistent) have any comorbidities at baseline. For that, the RFC trained on the combintation of all domains and all patients of the training set was evaluated within the two subgroups on the test set separately. The RFC obtained an AUC of 0.62 within the no-comorbidity group and an AUC of 0.73 within the comorbidity group. As in the case of the prediction of recovery from anxiety disorders the RFC was showing better performance for patients with comorbidities at baseline.

**Table 4.** Evaluation of the 2-year recovery from all common mental disorders classification [mean (s.d.)]

Domains	AUC	Accuracy	Sensitivity	Specificity	PPV	NPV
Clinical	0.70 (0.05)*	62.2 (4.6)	65.0 (7.1)	59.3 (5.6)	0.52 (0.05)	0.71 (0.05)
Psychological	0.67 (0.05)*	62.2 (4.8)	61.8 (8.4)	62.6 (6.4)	0.53 (0.05)	0.71 (0.05)
Socio-demographic	0.65 (0.05)*	60.8 (5.2)	65.2 (7.5)	56.5 (6.5)	0.51 (0.05)	0.70 (0.05)
Biological	0.57 (0.05)	56.0 (4.8)	57.5 (8.2)	54.6 (6.7)	0.47 (0.05)	0.65 (0.05)
Lifestyle	0.53 (0.05)	51.8 (4.7)	62.3 (7.9)	41.2 (6.5)	0.42 (0.04)	0.61 (0.06)
Combination	0.70 (0.05)*	63.4 (4.8)	64.6 (7.3)	62.3 (6.1)	0.54 (0.05)	0.72 (0.05)

AUC, area-under-receiver-operator-curve; PPV, positive predictive value; NPV, negative predictive value; *p_Bonferroni_ < 0.05.

*p* values shown in bold are <0.05.

#### Domain comparisons

The best performing domains for this classification were the same as in the recovery from anxiety disorders classification. The clinical domain and the combination of all domains did not differ in their performance but outperformed any other domain during the classification. The order for the performance of the other domains was the same as with the recovery from anxiety disorders classification.

#### Variable importance

48 variables were identified as being consistently selected significant variables contributing to the classification (online Supplementary Table 3). In this classification, selected variables included a larger set of measures related to mood disorders and not only anxiety symptomatology. With one exception (sociodemographic) all variables were again selected from the clinical or psychological domain.

### Difference in important variables between prediction analyses

Variables which were more (or less) important in the prediction of recovery from anxiety disorders than the prediction of all CMDs are reported in online Supplementary Table 4. These results confirmed the importance of anxiety-related variables for the prediction of recovery from anxiety, and the importance of depression-related variables for the prediction of recovery from all CMDs.

### Transfer analysis

We replicated the classification of recovery from anxiety disorders at 2-year follow-up in a transfer learning setting: in such an approach we utilized the labels indicating recovery of CMDs during the training of the RFC classifier (training set) but subsequently evaluated its performance on the test set using the recovery from anxiety disorder labels. The result of this analysis can be seen in online Supplementary Table 5. Utilizing the transfer learning approach led to improved performance in predicting anxiety disorder recovery (AUC = 0.71 *v.* AUC = 0.67 for both training and testing on anxiety disorder recovery labels using either only the clinical or the combination of all domains). The increased performance was observed due to an increase in sensitivity of the classification for correctly identifying recovered anxiety patients. For all individual domains and the combination of them, sensitivity increased by 7.6 ± 1.9 when training on the CMDs labels first. Specificity only decreased slightly (mean decrease: 2.7 ± 0.8) which led to the improved overall performance.

## Discussion

One of the most important goals in personalized medicine is providing individual disease course predictions. Our results show that individual prediction of 2-year course in anxiety disorders is possible using various predictors but it is only moderately successful. The main outcome measure was recovery from anxiety disorders and our predictions reached a balanced accuracy of 62.4% with an AUC of 0.67. The current performance by itself does not warrant implementation of our models in routine psychiatric care as it would yield too many false positives/negatives. However, predictive properties of clinician opinion in predicting disease course in anxiety disorders are not available and therefore it remains unclear which predictive performance threshold is needed for a statistical model to surpass clinician opinion and become an improvement over current routine care.

Our study yielded two models with comparable accuracy for predicting 2-year anxiety disorder course: one consisting of predictors from all five domains and one consisting of predictors only from the clinical domain. Biological, lifestyle, and sociodemographic predictors did not contribute significantly to course prediction. This is surprising as these domains were previously shown to be related to anxiety disorder aetiology. Our results thereby suggest that the underlying aetiology is of less importance to course prediction after the development of threshold disorders and that after anxiety disorders have developed, phenotypical characteristics have more impact on subsequent disease course. This is evident from the individual features that contributed most to the classification. All of these features reflected symptoms, psychological states or traits associated with the emotions of fear and anxiety, such as the presence of ‘phobic symptoms’, difficulty ‘walking alone in a busy street’ or ‘dealing with people you don't know’, ‘feeling tense’, ‘not liking to be where the action is’, and ‘feeling faint or lightheaded’. A previous NESDA study that aimed to predict the naturalistic course in depression showed similar performance to the current study when 2-year follow-up MDD diagnosis was correctly classified with an AUC of 0.66 and balanced accuracy of 62% (Dinga et al., 2018). In this study, clinical features were most important as well, though the nature of those items was related to depression.

As anxiety disorders and other psychiatric disorders frequently co-occur and show diagnostic instability over time, a secondary outcome was assessed. This broad perspective model was trained on recovery from all CMDs and showed marginally higher accuracy (63.4%) and AUC (0.70) in comparison with the main narrow perspective outcome. Like in the narrow perspective, omitting all domains except the clinical domain did not lead to a significant loss of predictive power (accuracy = 62.2% and AUC = 0.70). The individual features that were most consistently chosen during the classification again were almost exclusively from the clinical and psychological domains. Symptoms, psychological traits, and psychological states associated with depression and worrying contributed most to the classification. For instance: ‘feeling down’, ‘feeling sad’, having ‘a desire to die’, ‘suffering from worry’, ‘feeling tense’, and ‘having little control about the things that happen’. This suggests that predictions for recovery from all CMDs were largely driven by co-occurring depressive symptoms. Our decision to investigate the CMDs classification was also supported by the results of the additional transfer analysis which showed improved performance (accuracy = 63.3% and AUC = 0.71 for the combination of all domains data) when using the recovery from all CMDs labelling during training and the recovery from anxiety labels during model evaluation. This analysis showed that patients suffering from any mental disorder at 2-year follow-up – anxiety or not – constituted a more homogenous group while patients who fully recovered were more easily identified than patients only recovering from anxiety disorders (but having an additional CMD instead). This suggests that applying a broad perspective in future attempts in clinical prediction is more feasible for anxiety disorders.

Previous ML studies in anxiety disorders were invariably small in sample size and most focused on predicting immediate treatment response using neuroimaging data (Ball, Stein, Ramsawh, Campbell-Sills, & Paulus, 2014; Doehrmann et al., 2013; Hahn et al., 2014; Pantazatos, Talati, Schneier, & Hirsch, 2014; Whitfield-Gabrieli et al., 2016). Some studies used clinical, biological and/or neuroimaging data to distinguish between different types of anxiety disorders and healthy controls (Carpenter, Sprechmann, Calderbank, Sapiro, & Egger, 2016; Frick et al., 2014; Hilbert, Lueken, Muehlhan, & Beesdo-Baum, 2017; Pantazatos et al., 2014). To the best of our knowledge, this is the first study into individual long-term course prediction in anxiety disorders. A strength of this study is the use of a large dataset with a high number of variables from a variety of predictor domains, most of which were previously related to disease course at the group level. In addition, using RFCs allowed for combining large numbers of predictors into an overall model and allowed the identification of the most contributing predictors, providing insight into the possible processes involved with recovery in anxiety disorders.

In spite of the wide array of predictors, the current study showed only moderate accuracy. This has a number of explanations. First, NESDA is a naturalistic cohort study in which the exposure to environmental stressors and treatment regimens varied across patients during the 2-year follow-up period. These different exposures will have impacted the 2-year outcomes. Furthermore, different data types might improve predictive accuracy. For instance, previous ML studies showed the strong potential of neuroimaging data to predict treatment response in anxiety disorders (Ball et al., 2014; Doehrmann et al., 2013; Hahn et al., 2014; Pantazatos et al., 2014; Whitfield-Gabrieli et al., 2016), sometimes exceeding predictions made using clinical data (Ball et al., 2014; Doehrmann et al., 2013). Our study did not encompass neuroimaging data, as these were only available in a subset of NESDA participants (Janssen, Mourão-Miranda, & Schnack, 2018). Other examples include gait analysis (Zhao et al., 2019), actigraphy (Merikangas et al., 2019), or social media data (Reece & Danforth, 2017). Additionally, more frequent data collection might improve predictive accuracy (Kubben, Dumontier, & Dekker, 2019), which has now been implemented in the most recent wave of NESDA (Difrancesco et al., 2019). However, it is worth noting that our analyses showed that using a large set of variables from various domains (either combined or independently) did not outperform the clinical domain alone. Finally, future studies could explore differences in predictive performance across different patient subgroups, by analyzing separate patient groups consisting of different anxiety disorders, or groups with different comorbidity patterns separately.

Clinical care for anxiety disorders would benefit greatly from improved course prediction as it would pave the way for targeted treatments. The current study showed moderate accuracy in predicting recovery from anxiety disorders over a 2-year follow-up for individual patients. Items from the clinical and psychological domain were the most contributing predictors, while biological, lifestyle, and sociodemographic predictors were contributing less. The limited performance while using a wide array of predictors does not justify application in routine clinical care. The results from our study can, however, be used as a benchmark for future studies, with future studies likely resulting in further enhancements of the predictive properties. It has long been argued that statistical modelling will exceed clinician opinion in prediction problems (Ayres, 2007; Meehl, 1954), with clinician interpretation of statistical models likely yielding the best predictive power (Kuhn & Johnson, 2013). As a result, statistical models will increasingly become an addition to clinician opinion. Eventually, targeted treatment regimens and secondary prevention strategies will become more feasible if predictive models further evolve. This study provides an important first step towards valid long-term ML-based predictions in anxiety disorders.

## Supplementary material

For supplementary material accompanying this paper visit https://doi.org/10.1017/S0033291720001658. click here to view supplementary material
