# Supplementary material for: Predicting the naturalistic course in anxiety disorders using clinical and biological markers: a machine learning approach
Source: Psychol Med. 2020 Jun 11;52(1):57–67. doi: 10.1017/S0033291720001658 (PMC8711102; doi:10.1017/S0033291720001658)
Supplement: Supplementary file 1 [file S0033291720001658sup001.docx]

**Supplementary Information**

Predicting the naturalistic course in anxiety disorders using clinical and biological markers: a machine learning approach

Wicher A. Bokma**§***^1,2^* MD, Paul Zhutovsky**§***^3^* MSc, Erik J. Giltay*^4^* MD, PhD, Robert A. Schoevers*^5^* MD, PhD, Brenda W.J.H. Penninx*^1,2^* PhD, Anton L.J.M. van Balkom*^1,2^* MD, PhD, Neeltje M. Batelaan***§****^1,2^* MD, PhD, Guido A. van Wingen***§****^3^* PhD

*^1^ Department of Psychiatry, Amsterdam UMC, Vrije Universiteit, Amsterdam Public Health research institute, The Netherlands.*

*^2^ GGZ inGeest Specialized Mental Health Care, Amsterdam, The Netherlands.*

*^3^ Department of Psychiatry, Amsterdam UMC, Location AMC, University of Amsterdam, Amsterdam Neuroscience, Amsterdam, The Netherlands.*

*^4^ Department of Psychiatry, Leiden University Medical Center (LUMC), Leiden, The Netherlands.*

*^5^ Department of Psychiatry, University Medical Center Groningen, Groningen, The Netherlands.*

***§ both authors contributed equally***

**Supplementary Methods**

*Full descriptions of clinical domain measurement instruments*

Lifetime history for Anxiety and Depressive Disorders and presence of DSM-IV Panic Disorder, Panic Disorder with Agoraphobia, Agoraphobia, Generalized Anxiety Disorder, Social Anxiety Disorder, MDD, Dysthymia and Alcohol Dependency (one month, six-month, one year recency) at baseline were assessed using the WHO-Composite International Diagnostic Interview (CIDI, version 2.1). The CIDI is a structured interview with good reliability and validity (World Health Organization 1998).

Sleep quality during the past four weeks was assessed with the Women’s Health Initiative Insomnia Rating Scale (IRS) (Levine *et al.* 2003). This questionnaire consists of six items and a summary score.

Mood and anxiety symptoms during the past week were assessed with the Mood and Anxiety Scoring Questionnaire (Watson *et al.* 1995). The MASQ consists of 30 individual items and three summary scores: positive affect, negative affect and levels of somatization.

Presence of lifetime bipolar symptoms were assessed with the Mood Disorder Questionnaire (MDQ) (Hirschfeld *et al.* 2000). This questionnaire consists of 13 individual items and a summary score.

Levels of general distress and somatization during the past week were measured with the Four-Dimensional Symptom Questionnaire (4DSQ) (Terluin 1996). These two dimensions were measured in 32 items and two summary scores.

Levels of pathological worrying tendencies were assessed using the 11-item self-report version of the Penn State Worry Questionnaire (PSWQ) (Drost *et al.* 2012).

Depressive symptoms during the past week were assessed with the Inventory of Depressive Symptomatology-SR (IDS-SR) (Rush *et al.* 1986, 1996). The IDS-SR consists of 30 individual items and a summary score.

Symptoms of anxiety during the past month were assessed with the Beck Anxiety Inventory (BAI), a 21-item self-report questionnaire (Beck *et al.* 1988).

Presence of avoidance behaviors was assessed with the Fear Questionnaire (FQ), a 15-item self-report questionnaire (Marks & Mathews 1979).

Presence of childhood trauma was assessed with a 3-item adaptation from the NEMESIS questionnaire (Wiersma *et al.* 2009).

Symptoms of suicidality during the past week were assessed with the 5 items from the Suicidal Ideation Scale (SSI) that refer to the current state (Beck *et al.* 1979).

The total number of negative life-events during the past year was assessed with the Brugha questionnaire (Brugha *et al.* 1985).

Duration of anxiety symptoms during the last four years prior to baseline were assessed with the Life Chart Interview (LCI), a structured retrospective interview using a calendar approach (Lyketsos *et al.* 1994). The LCI has adequate reliability and validity (Warshaw *et al.* 1994). Due to large proportions of missingness, time spent with depressive and avoidance symptoms were not included in analyses.

Subjects’ convictions about the importance of care and their past experiences with care in relation to mental health problems were assessed with the 36-item QUality Of care Through the Eyes of the patient (QUOTE): Anxiety/Depression version (Sixma *et al.* 1998).

Subjects’ perceived need for care at various domains during the past six months was assessed with the 20-item Perceived Need for Care Questionnaire (PNCQ) (Meadows *et al.* 2000). After missing data handling, 14 items were included in analyses.

Levels of disability were measured with the WHO-Disability Assessment Schedule (WHO-DAS II), a 36-item self-report questionnaire measuring levels of disability (Chwastiak & Von Korff 2003). Four work-related items were omitted from further analyses due to large proportions of missing values.

All baseline pharmacotherapeutic use was assessed using inspection of medication containers and coded according to Anatomical Therapeutic Chemical (ATC) codes at baseline (WHO Collaborating Centre for Drug Statistics Methodology n.d.). Historic use of psychopharmacotherapeutics during the three-year period prior to baseline was reported retrospectively.

*Full descriptions of psychological domain measurement instruments*

Anxiety sensitivity was measured with the 16-item Anxiety Sensitivity Index (ASI) (Reiss *et al.* 1986).

Cognitive reactivity to sadness was measured with the 34-item Leiden Index of Depression Sensitivity (LEIDS) (Van der Does 2002).

Levels of mastery were assessed with an adapted version of the Pearlin Mastery Scale, consisting of 5 items (Pearlin & Schooler 1978; Kempen *et al.* 1998).

Personality structure was assessed with the NEO Five-Factor Inventory, a shortened version of the Revised NEO Personality Inventory (McCrae & Costa 2004). In our analyses, all 60 individual items, as well as domain scores from the five domains were used.

In order to be able to assess relative levels of these psychological traits, summary scores were standardized at the level of the whole NESDA sample.

*Full descriptions of sociodemographic domain measurement instruments*

Information on sociodemographic information from subject was gathered in a structured manner by face-to-face interviews with trained research assistants. Information gained referred to demographic characteristics (6 items), employment status (6 items), marital status (2 items, of which one was omitted due to high number of missing values), sexual preference (2 items, of which one was omitted due to high number of missing values), housing status (5 items), sources and level of income (11 items), religion status (3 items of which two were omitted due to high number of missing values), family and household decomposition (23 items, of which seventeen were omitted due to high number of missing values), and participation in various leisure activities (23 items, of which three were omitted due missing data). Employment status was analyzed categorically, but presented in the descriptive statistics table dichotomously. Categories that we presented as ‘currently employed’ included ‘now employed’, ‘self employed’ and ‘on pregnancy or maternity leave’. The remaining categories were ‘occupationally disabled’, ‘on sickness benefit’, ‘early retirement’, ‘unemployed’ and ‘other’.

Current levels of loneliness were assessed with the de Jong-Gierveld loneliness scale, an 11 item self-report questionnaire (de Jong-Gierveld & Kamphuls 1985). Severe loneliness was defined as a maximum score of 11.

Levels of current social support were assessed with the 38-item Close Person Inventory (CPI) (Stansfeld & Marmot 1992). However, due to large proportions of missing data, only 3 items were included in our analyses.

*Full descriptions of biological domain measurement instruments*

The number of chronic diseases with or without treatment were assessed using a 21-item face-to-face interview (Penninx *et al.* 2008). Subjects were asked for presence of 30 common chronic somatic diseases and were able to report any additional diseases they may have. This yielded 2 items that were both included.

Levels of chronic pain during the past 6 months were assessed with the chronic graded pain scale in which levels of chronic pain are summarized in a single ordinal item consisting of 5 grades of pain (Von Korff *et al.* 1992).

The current menstrual cycle status was assessed in five self-reported items, of which 4 were used in analyses.

Body Mass Index (BMI) and hip/waist circumference ratio were measured by a trained research assistant, in accordance to international standards.(World Health Organisation 1989)

Autonomic nervous system function was reflected by measurements of mean heart rate, heart rate variability, inter-beat-interval, pre-ejection period, aggregated respiratory sinus arrhythmia, and aggregated respiration rate. These were measured during the baseline data collection interview with the Vrije Universiteit Ambulatory Measuring System (Vu-AMS) (de Geus *et al.* 1995).

Systolic and diastolic blood pressure was measured with the OMRON M4 IntelliSense digital blood pressure monitor (*HEM-752A, Omron Healthcare, Inc., Bannockburn, Illinois, USA* n.d.). In NESDA, the average of two measurements was used.

Handedness was assessed by self-report. Hand-grip strength, a proxy for overall muscle strength, was assessed twice with the Jamar dynamometer (Bellace *et al.* 2000; Ashton & Myers 2004).

Whether subjects had a fever or a cold during the last week was assessed by self-report.

Fasting blood samples of NESDA participants were obtained in the morning around 8 am and kept frozen at -80°C. Various laboratory tests were performed on these samples.

Brain-Derived Neurotropic Factor (BDNF), Triglycerides, High Density Cholesterol (HDL), glucose, tryptophan, kynurenine, 3-Hydroxykynurenine,

Cystatin C, Urea, Uric acid, Creatinin, Cotinine, Parathyroid hormone (PTH), 25-hydroxy vitamin D, Dehydroepiandrosterone, Dehydroepiandrosterone sulfate (DHEA-S), Sex hormone-binding globulin (SHBG), Estradiol (E2), and Testosterone (nmol/l) were assayed at the Clinical Chemistry department of the VU University Medical Center using standard laboratory procedures. Dehydroepiandrosterone measurements were omitted due to missings.

High-sensitivity plasma levels of C-reactive protein (CRP) were measured in duplicate by an in-house ELISA based on purified protein and polyclonal anti‑CRP antibodies.(*Dako, Glostrup, Denmark* n.d.) The CRP assay was standardized against the CRM 470 reference agent. The lower detection limit of CRP was 0.1 mg/l and the sensitivity was 0.05 mg/l.

Plasma Interleukine-6 (IL-6) levels were measured in duplicate by a high sensitivity enzyme-linked immunosorbent assay.(*PeliKine CompactTM ELISA, Sanquin, Amsterdam, The Netherlands* n.d.) The IL-6 assay was standardized against a recombinant human IL-6 standard. The lower detection limit of IL-6 was 0.35 pg/ml and the sensitivity 0.10 pg/ml.

Plasma Tumour necrosis factor-alpha (TNF-α) levels were assayed in duplicate at Good Biomarker Science, Leiden, The Netherlands, using a high-sensitivity solid phase ELISA.(*Quantikine® HS Human TNF- α Immunoassay, R&D systems Inc, Minneapolis, MN, United States* n.d.) The TNF-α assay was calibrated against a highly purified E. coli-expressed recombinant human TNF-α. The lower detection limit of TNF-α was 0.10 pg/ml and the sensitivity 0.11 pg/ml.

*Full descriptions of lifestyle domain measurement instruments*

Smoking status was assessed with three items from the Fagerström Test for Nicotine Dependence (Heatherton *et al.* 1991). Subjects were divided into current smokers, former smokers and subjects who never smoked. Two items were omitted due to missings.

Number of different psychoactive banned substances used by subjects was assessed by self-report.

The amount of alcohol consumption during the past year was assessed with the Alcohol Use Disorders Identification Test (AUDIT) (Saunders *et al.* 1993).

The levels of physical exercise, expressed in metabolic equivalent of task (MET)-minutes/week during the past week were assessed with the 4-item International physical activity questionnaire (Ainsworth *et al.* 2000).

*Extended description random forest classifier*

Each Random Forest classifier (RFC) was build using 1000 classification trees (Breiman *et al.* 1984) and the number of randomly selected variables per node was set at the square root of the number of variables (default value). Subsample aggregating (subagging) was used instead of bootstrap aggregating (bagging) to create new random subsets of data points per tree. Subagging allowed for balancing the data set (Chen *et al.* 2004) by sampling the same number of subjects for each class, and improving the validity of variable importance calculations (Strobl *et al.* 2007). The balancing of the classes can improve classification performance in data sets with imbalanced distribution of classes where a classifier might focus on only correctly predicting the majority class by assigning all data to this class. 63.2% of all subjects was used as the subsampling factor. This corresponds to the number of unique subjects in a bootstrap sample when using bagging and is recommended as a default (Boulesteix *et al.* 2012).

*Variable importance calculation*

The standard calculation of variable importance for RFC has been shown to be biased towards continuous variables and categorical variables with many categories (Strobl *et al.* 2007). To ensure the validity of variable importance calculations it was suggested to use subagging and permutation-based variable importance calculations (Strobl *et al.* 2007; Altmann *et al.* 2010; Hapfelmeier & Ulm 2013). To implement permutation-based variable importance calculations we permuted each variable separately a 1000 times and assessed its variable importance under permutation (Ojala & Garriga 2010). The computed null-distribution was then used to calculate a *P*-value of the actually observed variable importance for each variable.

*Difference in selected variables*

To compare whether variable importance differed between the two classification tasks the following analysis was conducted: 1. based on the *P*-values calculated from permutation-based variable importance we computed a rank from most important (smallest *P*-value) to least important variable per cross-validation iteration, 2. we averaged this ranking across the cross-validation runs to obtain an average rank for each variable, 3. we calculated the absolute difference of ranks between the two classification tasks, 4. we explored each rank difference which was higher (lower) than the mean rank difference +(-) twice the standard deviation. In this way we could determine which variables average rank changed strongly between the two different classification tasks.

**Supplementary Table 1.** Baseline characteristics of anxiety disorder sample, group comparisons between patients who had no common mental disorders (n = 362) at two-year follow-up and patients who did have any common mental disorder at follow-up (n = 525)

| **Two-year common mental disorders status**  **Baseline characteristics** | **Recovered  (n = 362)** | **Persistent (n = 525)** | **Statistics** | **p** |
| --- | --- | --- | --- | --- |
| **Clinical domain** |  |  |  |  |
| PD diagnosis | 135 (37.3%) | 233 (44.4%) | Χ^2^ = 4.34 | **0.035** |
| Agoraphobia diagnosis | 111 (30.7%) | 206 (39.2%) | Χ^2^ = 6.86 | **0.009** |
| SAD diagnosis | 147 (40.6%) | 261 (49.7%) | Χ^2^ = 7.15 | **0.007** |
| GAD diagnosis | 92 (25.4%) | 185 (35.2%) | Χ^2^ = 9.63 | **0.002** |
| MDD diagnosis | 103 (28.5%) | 259 (49.3%) | Χ^2^ = 38.7 | **<0.001** |
| Dysthymia diagnosis | 26 (7.2%) | 120 (22.9%) | Χ^2^ = 38.3 | **<0.001** |
| Use of psychotropic medication, *current* | 252 (69.6%) | 387 (73.7%) | Χ^2^ = 1.79 | 0.181 |
| Avoidance behaviour severity, *mean* *FQ*, *current* | 30.96 ± 18.64 | 39.33 ± 20.06 | t = -6.37 | **<0.001** |
| Pathological worrying severity,   *mean* *PSWQ*, *current* | 35.05 ± 9.81 | 39.35 ± 9.47 | t = -6.49 | **<0.001** |
| Suicidal thoughts, *SSI*, *past week* | 38 (10.5%) | 145 (27.6%) | Χ^2^ = 38.6 | **<0.001** |
| Level of distress, *mean* *4DSQ*, *past week* | 14.78 ± 8.79 | 19.83 ± 8.66 | t = 8.47 | **<0.001** |
| Depressive symptoms severity,   *mean* *IDS-SR*, *past week* | 24.96 ± 11.82 | 32.47 ± 12.92 | t = -9.14 | **<0.001** |
| Sleep disturbances, *mean* *ISR,* *past four weeks* | 9.05 ± 4.94 | 10.23 ± 5.33 | t = -3.38 | **0.001** |
| Anxiety symptoms severity,   *mean* *BAI*, *past month* | 15.57 ± 9.40 | 20.19 ± 10.79 | t = -6.77 | **<0.001** |
| Percentage of time spent with anxiety symptoms, *LCI, past four years* | 42.4% ± 32.9 | 52.7% ± 34.2 | t = -4.36 | **<0.001** |
| History of childhood life events^1^ | 68 (18.8%) | 95 (18.1%) | Χ^2^ = 0.07 | 0.79 |
| History of childhood trauma^2^ | 182 (50.3%) | 323 (61.6%) | Χ^2^ = 11.3 | **0.001** |
| History of serious suicide attempts | 43 (11.9%) | 110 (21.0%) | Χ^2^ = 12.1 | **<0.001** |
| **Psychological domain** |  |  |  |  |
| Neuroticism, *mean* *NEO-FF subscale* | 39.77 ± 6.88 | 43.39 ± 6.69 | t = -7.78 | **<0.001** |
| Extraversion,  *mean* *NEO-FFI subscale* | 35.40 ± 6.44 | 32.40 ± 6.67 | t = 6.93 | **<0.001** |
| Conscientiousness,  *mean* *NEO-FFI subscale* | 41.54 ± 6.19 | 39.16 ± 6.46 | t = 5.52 | **<0.001** |
| Agreeableness, *mean NEO-FFI subscale* | 43.84 ± 5.13 | 42.46 ± 5.41 | t = 3.87 | **<0.001** |
| Openness,  *mean NEO-FFI subscale* | 38.13 ± 5.99 | 38.18 ± 6.28 | t = -0.13 | 0.90 |
| Cognitive reactivity to sadness, *mean* *LEIDS* | 38.77 ± 17.91 | 46.77 ± 17.71 | t = -6.55 | **<0.001** |
| Anxiety sensitivity, *mean* *ASI* | 33.45 ± 9.63 | 36.02 ± 10.16 | t = -3.77 | **<0.001** |
| Mastery, *mean Mastery scale* | 16.49 ± 3.83 | 13.82 ± 4.00 | t = 9.97 | **<0.001** |
| **Sociodemographic domain** |  |  |  |  |
| Age in years | 40.94 ± 12.25 | 42.59 ± 12.13 | t = -1.97 | **0.047** |
| Education years | 12.16 ± 3.29 | 11.68 ± 3.37 | t = 2.12 | **0.034** |
| Female gender | 252 (69.6%) | 353 (67.2%) | Χ^2^ = 0.56 | 0.45 |
| Currently employed | 218 (60.2%) | 268 (51.0%) | Χ^2^ = 7.28 | **0.007** |
| Has children | 168 (46.4%) | 239 (45.5%) | Χ^2^ = 0.07 | 0.80 |
| Current severe loneliness | 29 (8.0%) | 76 (14.5%) | Χ^2^ = 8.50 | **0.004** |
| **Biological domain** |  |  |  |  |
| Number of chronic somatic diseases | 0.61 ± 0.84 | 0.75 ± 0.96 | t = -2.25 | **0.025** |
| Chronic pain with high disability | 67 (18.5%) | 154 (29.3%) | Χ^2^ = 13.4 | **<0.001** |
| BMI | 25.28 ± 4.64 | 25.77 ± 5.38 | t = -1.47 | 0.14 |
| Mean heart rate (bpm) | 72.03 ± 9.76 | 71.75 ± 9.89 | t = 0.40 | 0.69 |
| Systolic blood pressure (mmHg) | 135.7 ± 20.91 | 136.5 ± 18.4 | t = -0.62 | 0.53 |
| CRP (mg/L, n=876) | 2.70 ± 4.10 | 2.99 ± 5.83 | t = -0.88 | 0.38 |
| IL-6 (pg/ml, n=876) | 1.23 ± 3.05 | 1.43 ± 3.08 | t = -0.94 | 0.35 |
| TNF-α (pg/ml, n=871) | 1.09 ± 1.36 | 1.04 ± 1.09 | t = 0.55 | 0.58 |
| BDNF(ng/ml, n=865) | 9.26 ± 3.57 | 9.14 ± 3.54 | t = 0.48 | 0.63 |
| **Lifestyle domain** |  |  |  |  |
| Former smoker | 119 (32.9%) | 153 (29.1%) | Χ^2^ = 3.99 | 0.14 |
| Current smoker | 125 (34.5%) | 216 (41.1%) |  |  |
| Low physical activity, *past week* | 64 (18.9%) | 137 (27.3%) | Χ^2^ = 11.5 | **0.003** |
| High physical activity, *past week* | 129 (38.1%) | 144 (28.7%) |  |  |
| Any substance use, *past week* | 22 (6.1%) | 44 (8.4%) | Χ^2^ = 1.65 | 0.20 |
| Hazardous drinking or alcohol dependency^3^ *past year* | 76 (21.1%) | 120 (22.9%) | Χ^2^ = 0.38 | 0.54 |

PD: Panic Disorder; SAD: Social Anxiety Disorder; GAD: Generalized Anxiety Disorder; MDD: Major Depressive Disorder; FQ: Fear Questionnaire; PSWQ: Penn State Worry Questionnaire; SSI: Suicidal Ideation Scale; 4DSQ: Four Dimensional Symptom Questionnaire; IDS-SR: Inventory of Depressive Symptomatology-SR; ISR: Insomnia Rating Scale; BAI: Beck’s Anxiety Inventory; LCI: Life chart interview; NEO-FFI: NEO Five-Factor Inventory; LEIDS: Leiden Index of Depression Sensitivity; ASI: Anxiety Sensitivity Index; BMI: Body Mass Index; CRP: c-reactive protein; IL-6: interleukin-6; TNF-α: tumor necrosis factor-α; BDNF: Brain Derived Neurotrophic Factor.

^1^ childhood life events (<16 years of age) were parental divorce, being placed in a juvenile prison, raised in a foster family, placed in a child home, death of a parent.

^2^ childhood trauma included emotional neglect, psychological abuse, physical abuse and sexual abuse
^3^ as measured with the AUDIT. Scores above 8 are reflective of hazardous drinking, scores at 13 or higher (females) and 15 or higher (males) are indicative of probable alcohol dependency.

**Supplementary Table 2:** Consistently selected significant variables in the recovery from anxiety disorders classification

| **Item** | **Description** | **Selection Frequency [%]** |
| --- | --- | --- |
| NEO-FFI item 31 | I rarely feel fearful or anxious | 98 |
| IDS-SR item 27 | Panic/Phobic symptoms | 98 |
| WHO DAS item 38 | How much embarrassment did you experience because of your health problems during the past 30 days? | 97 |
| FQ item 05 | Walking alone in a busy street | 95 |
| CIDI PDA 1m | Panic with agoraphobia - past month | 94 |
| Life chart item 01 | percent of time with anxiety symptoms | 87 |
| WHO DAS item 16 | Dealing with people you do not know? | 83 |
| NEO-FFI, neuroticism | Anxiety alternative rationally derived decomposition of neuroticism domain | 78 |
| CIDI PDA 12m | Panic with agoraphobia - past year | 77 |
| IDS-SR item 07 | Feeling Anxious or Tense | 74 |
| 4DSQ item 05 | During the past week did you feel: tense? | 70 |
| NEO-FFI, item 22 | I like to be where the action is | 69 |
| MASTERY item 04 | I often feel helpless dealing with the problems of life | 66 |
| CIDI SAD l | Social Anxiety Disorder - in lifetime | 63 |
| CIDI PDA 6m | Panic with agorafobia - past 6 months | 61 |
| BAI item 19 | Faint, lightheaded | 59 |
| 4DSQ item 02 | During the past week did you suffer from: worry? | 54 |

**Supplementary Table 3:** Consistently selected significant variables in the recovery from common mental disorders classification

| **Variable Name** | **Description** | **Selection Frequency [%]** |
| --- | --- | --- |
| MASQ item 03 | Felt successful | 100 |
| 4DSQ item 01 | During the past week did you suffer from: feeling down or depressed? | 100 |
| CIDI dysthymia 1m | Dysthymia  - past month | 100 |
| IDS-SR item 05 | Feeling Sad | 100 |
| 4DSQ item 11 | During the past week did you feel: that you can’t enjoy anything anymore? | 100 |
| CIDI dysthymia 6m | Dysthymia  - past 6 months | 100 |
| 4DSQ item 09 | During the past week did you feel: that you can’t cope anymore? | 100 |
| 4DSQ item 08 | During the past week did you feel: that you can no longer take interest in the people and things around you? | 100 |
| 4DSQ item 02 | During the past week did you suffer from: worry? | 100 |
| 4DSQ item 10 | During the past week did you feel: that you can’t face it anymore? | 100 |
| Mastery item 02 | Some of my problems I cannot seem to solve at all | 100 |
| NEO-FFI, neuroticism | Selfreproach | 100 |
| Mastery item 04 | I often feel helpless dealing with the problems of life | 98 |
| Mastery item 05 | Sometimes I feel like a play ball of life | 98 |
| 4DSQ item 05 | During the past week did you feel: tense? | 97 |
| CIDI MDD 12m | Major Depression - past year | 97 |
| CIDI dysthymia 12m | Dysthymia  - past year | 96 |
| SSI item 02 | Desire to die | 95 |
| IDS-SR item 08 | Response of Your Mood to Good or Desired Events | 95 |
| Mastery item 01 | I have little control about the things that happen to me | 95 |
| IDS-SR item 07 | Feeling Anxious or Tense | 93 |
| CIDI MDD 1m | Major Depression - past month | 93 |
| MASQ item 25 | Had trouble making decisions | 91 |
| IDS-SR item 21 | Capacity for Pleasure or Enjoyment (excluding sex) | 90 |
| MASQ item 22 | Felt really "up" or lively | 88 |
| Life chart anxiety | percent of time with anxiety symptoms | 87 |
| BAI item 19 | Faint, lightheaded | 83 |
| MASQ item 06 | Felt really happy | 82 |
| MASQ item 13 | Felt dissatisfied with everything | 79 |
| MASQ item 11 | Felt like I was having a lot of fun | 79 |
| SSI item 01 | Desire to live | 76 |
| Loneliness item 03 | I experience a general sense of emptiness | 73 |
| IDS-SR item 12 | Quality of Your Mood | 72 |
| BAI item 08 | Unsteady | 70 |
| NEO-FFI, neuroticism | depression | 69 |
| IDS-SR item 15 | Concentration/Decision Making | 68 |
| MASQ item 23 | Felt inferior to others | 66 |
| MASTERY item 03 | There is not much that I can do to change important things in my life | 64 |
| MASQ item 29 | Felt really good about myself | 62 |
| MASQ item 28 | Worried a lot about things | 62 |
| QUOTE item 10 | Started feeling in control over my problems. | 60 |
| PSWQ item 02 | Many situations make me worry | 59 |
| NEO-FFI, conscientiousness | Orderliness | 59 |
| QUOTE item 16 | The professional taught me how to deal with future symptoms. | 54 |
| QUOTE item 05 | The general practicioner explained the pros and cons of different medications. | 53 |
| MASQ item 21 | Was short of breath | 53 |
| IDS-SR item 30 | Leaden Paralysis/Physical Energy | 52 |
| NEO-FFI, neuroticism | Negative affect | 51 |

**Supplementary Table 4:** Variables which were more (or less) important in the broad perspective (recovery from anxiety and affective disorders) in comparison to the narrow perspective (recovery from anxiety disorders).

| **Variable Name** | **Description** |
| --- | --- |
| **More important for narrow perspective** | |
| WHO DAS item 16 | Dealing with people you do not know |
| BAI item 21 | Hot, cold sweats |
| FQ item 04 | Traveling alone by train or bus |
| IDS-SR item 26 | Other bodily symptoms |
| BAI item 05 | Fear of worst happening |
| CIDI SAD l | Social Anxiety Disorder - in lifetime |
| Blood plasma item 02 | Kynurenine (µmol/l) |
| WHO DAS item 38 | How much embarrassment did you experience because of your health problems during the past 30 days? |
| CIDI SAD 12m | Social Anxiety Disorder - past year |
| **More important for broad perspective** | |
| loneliness item 03 | I experience a general sense of emptiness |
| QUOTE item 16 | The professional taught me how to deal with future symptoms |
| 4DSQ item 07 | During the past week did you feel: that you just can’t do anything anymore? |
| CIDI MDD 1m | Major Depression - past month |
| MASQ item 26 | Felt like I had a lot of energy |
| CIDI MDD 6m | Major Depression - past 6 months |
| MASQ item 10 | Felt hopeless |
| QUOTE item 10 | Started feeling in control over my problems. |
| SSI item 03 | Reasons for living or dying |
| CIDI dysthymia m12 | Dysthymia  - past year |
| MASQ item 13 | Felt dissatisfied with everything |
| CIDI MDD 12m | Major Depression - past year |
| QUOTE item 17 | The professional reduced my symptoms. |
| 4DSQ item 14 | During the past week: tingling in the fingers |
| 4DSQ item 06 | During the past week did you feel: easily irritated |
| IDS-SR item 02 | Sleep During the Night |
| 4DSQ item 04 | During the past week did you suffer from: listlessness? |

**Supplementary Table 5:** Evaluation of the two-year recovery from anxiety disorders classification using a transfer learning approach [mean (SD)]

| **Domains** | **AUC** | **Accuracy** | **Sensitivity** | **Specificity** | **PPV** | **NPV** |
| --- | --- | --- | --- | --- | --- | --- |
| **Clinical** | 0.71 (0.05) | 62.5 (5.3) | 65.4 (8.7) | 59.7 (7.3) | 0.53 (0.06) | 0.72 (0.06) |
| **Psychological** | 0.67 (0.05) | 62.0 (5.0) | 61.6 (8.6) | 62.4 (6.6) | 0.53 (0.05) | 0.70 (0.05) |
| **Socio- demographic** | 0.65 (0.06) | 60.6 (5.7) | 64.4 (8.7) | 56.7 (7.3) | 0.51 (0.06) | 0.70 (0.06) |
| **Biological** | 0.57 (0.05) | 55.4 (4.7) | 57.3 (7.8) | 53.6 (6.4) | 0.46 (0.04) | 0.65 (0.05) |
| **Lifestyle** | 0.53 (0.06) | 51.7 (4.5) | 62.0 (7.2) | 41.5 (6.9) | 0.42 (0.04) | 0.61 (0.06) |
| **Combination** | 0.71 (0.05) | 63.3 (4.8) | 65.0 (8.5) | 61.7 (6.0) | 0.54 (0.05) | 0.72 (0.05) |

AUC, area-under-receiver-operator-curve; PPV, positive predictive value; NPV, negative predictive value

**References**

**Ainsworth BE, Bassett Jr. DR, Strath SJ, Swartz AM, O’Brien WL, Thompson RW, Jones DA, Macera CA, Kimsey CD** (2000). Comparison of three methods for measuring the time spent in physical activity. *Medicine & Science in Sports & Exercise*

**Altmann A, Toloşi L, Sander O, Lengauer T** (2010). Permutation importance: A corrected feature importance measure. *Bioinformatics* **26**, 1340–1347.

**Ashton LA, Myers S** (2004). Serial Grip Strength Testing- Its Role In Assessment Of Wrist And Hand Disability. *The Internet Journal of Surgery* **5**

**Beck A, Epstein N, Brown G, Steer R** (1988). An inventory for measuring clinical anxiety: Psychometric properties. *Journal of consulting and Clinical Psychology* **56**, 893–897.

**Beck AT, Kovacs M, Weissman A** (1979). Assessment of suicidal intention: The Scale for Suicide Ideation. *Journal of Consulting and Clinical Psychology*

**Bellace J V., Healy D, Besser MP, Byron T, Hohman L** (2000). Validity of the Dexter Evaluation System’s Jamar dynamometer attachment for assessment of hand grip strength in a normal population. *Journal of Hand Therapy*

**Boulesteix AL, Janitza S, Kruppa J, König IR** (2012). Overview of random forest methodology and practical guidance with emphasis on computational biology and bioinformatics. *Wiley Interdisciplinary Reviews: Data Mining and Knowledge Discovery* **2**, 493–507.

**Breiman L, Friedman JH, Olshen RA, Stone CJ** (1984). *Classification and regression trees*. Wadsworth & Brooks/Cole Advanced Books & Software: Monterey, CA.

**Brugha T, Bebbington P, Tennant C, Hurry J** (1985). The List of Threatening Experiences: a subset of 12 life event categories with considerable long-term contextual threat. *Psychological Medicine* **15**, 189.

**Chen C, Liaw A, Breiman L** (2004). Using Random Forest to Learn Imbalanced Data. *University of California, Berkeley*

**Chwastiak LA, Von Korff M** (2003). Disability in depression and back pain: Evaluation of the World Health Organization Disability Assessment Schedule (WHO DAS II) in a primary care setting. *Journal of Clinical Epidemiology* **56**, 507–514.

***Dako, Glostrup, Denmark*** (n.d.).

**Van der Does W** (2002). Cognitive reactivity to sad mood: Structure and validity of a new measure. *Behaviour Research and Therapy*

**Drost J, Der Does AJW, Antypa N, Zitman FG, Van Dyck R, Spinhoven P** (2012). General, specific and unique cognitive factors involved in anxiety and depressive disorders. *Cognitive Therapy and Research* **36**, 621–633.

**de Geus EJC, Willemsen GHM, Klaver CHAM, van Doornen LJP** (1995). Ambulatory measurement of respiratory sinus arrhythmia and respiration rate. *Biological Psychology* **41**, 205–227.

**Hapfelmeier A, Ulm K** (2013). A new variable selection approach using Random Forests. . Elsevier B.V. *Computational Statistics and Data Analysis* **60**, 50–69.

**Heatherton TF, Kozlowski LT, Frecker RC, Fagerstrom K-O** (1991). The Fagerstrom Test for Nicotine Dependence: a revision of the Fagerstrom Tolerance Questionnaire. *Addiction* **86**, 1119–1127.

***HEM-752A, Omron Healthcare, Inc., Bannockburn, Illinois, USA*** (n.d.).

**Hirschfeld RMA, Williams JBW, Spitzer RL, Calabrese JR, Flynn L, Keck J, Lewis L, McElroy SL, Post RM, Rapport DJ, Russell JM, Sachs GS, Zajecka J** (2000). Development and validation of a screening instrument for bipolar spectrum disorder: The mood disorder questionnaire. *American Journal of Psychiatry*

**de Jong-Gierveld J, Kamphuls F** (1985). The Development of a Rasch-Type Loneliness Scale. *Applied Psychological Measurement* **9**, 289–299.

**Kempen GIJ, Brilman EI, Ormel J** (1998). The Groningen Longitudinal Aging Study (GLAS) on functional status, well-being and need of care: Objectives, design and preliminary results . *Tijdschrift voor Gerontologie en Geriatrie*

**Von Korff M, Ormel J, Keefe FJ, Dworkin SF** (1992). Grading the severity of chronic pain. *Pain* **50**, 133–149.

**Levine DW, Kaplan RM, Shumaker SA, Kripke DF, Bowen DJ, Naughton MJ, Lewis MA** (2003). Reliability and validity of Women’s Health Initiative Insomnia Rating Scale. *Psychological Assessment*

**Lyketsos CG, Nestadt G, Cwi J, Heithoff K, et al** (1994). *The Life Chart Interview: A standardized method to describe the course of psychopathology.* *International Journal of Methods in Psychiatric Research* **4**, 143–155.

**Marks IM, Mathews AM** (1979). Brief standard self-rating for phobic patients. *Behaviour Research and Therapy* **17**, 263–267.

**McCrae RR, Costa PT** (2004). A contemplated revision of the NEO Five-Factor Inventory. *Personality and Individual Differences* **36**, 587–596.

**Meadows G, Harvey C, Fossey E, Burgess P** (2000). Assessing perceived need for mental health care in a community survey: development of the Perceived Need for Care Questionnaire (PNCQ). *Social psychiatry and psychiatric epidemiology* **35**, 427–35.

**Ojala M, Garriga GC** (2010). Permutation Tests for Studying Classifier Performance. *Journal of Machine Learning Research* **11**, 1833–1863.

**Pearlin LI, Schooler C** (1978). The Structure of Coping. *Journal of Health and Social Behavior* **19**, 2.

***PeliKine CompactTM ELISA, Sanquin, Amsterdam, The Netherlands*** (n.d.).

**Penninx BWJH, Beekman ATF, Smit JH, Zitman FG, Nolen WA, Spinhoven P, Cuijpers P, De Jong PJ, Van Marwijk HWJ, Assendelft WJJ, Van Der Meer K, Verhaak P, Wensing M, De Graaf R, Hoogendijk WJ, Ormel J, Van Dyck R** (2008). The Netherlands Study of Depression and Anxiety (NESDA): rationale, objectives and methods. *International journal of methods in psychiatric research* **17**, 121–40.

***Quantikine® HS Human TNF- α Immunoassay, R&D systems Inc, Minneapolis, MN, United States*** (n.d.).

**Reiss S, Peterson RA, Gursky DM, McNally RJ** (1986). Anxiety sensitivity, anxiety frequency and the predictions of fearfulness. *Behaviour Research and Therapy* **24**, 1-8 ST-Anxiety sensitivity, anxiety frequency a.

**Rush AJ, Giles DE, Schlesser MA, Fulton CL, Weissenburger J, Burns C** (1986). The inventory for depressive symptomatology (IDS): Preliminary findings. *Psychiatry Research* **18**, 65–87.

**Rush AJ, Gullion CM, Basco MR, Jarrett RB, Trivedi MH** (1996). The Inventory of Depressive Symptomatology (IDS): psychometric properties. *Psychological medicine* **26**, 477–486.

**Saunders JB, Aasland OG, Babor TF, De La Fuente JR, Grant M** (1993). Development of the Alcohol Use Disorders Identification Test (AUDIT): WHO Collaborative Project on Early Detection of Persons with Harmful Alcohol Consumption-II. *Addiction* **88**, 791–804.

**Sixma HJ, Kerssens JJ, Campen C van, Peters L** (1998). Quality of care from the patients’ perspective: from theoretical concept to a new measuring instrument. *Health Expectations* **1**, 82–95.

**Stansfeld S, Marmot M** (1992). Deriving a survey measure of social support: The reliability and validity of the close persons questionnaire. *Social Science & Medicine* **35**, 1027–1035.

**Strobl C, Boulesteix A-L, Zeileis A, Hothorn T** (2007). Bias in random forest variable importance measures: Illustrations, sources and a solution. *BMC Bioinformaticsoot* **8**, 1–21.

**Terluin B** (1996). De Vierdimensionale Klachtenlijst (4DKL) - Een vragenlijst voor het meten van distress, depressie, angst en somatisatie. [The Four-Dimensional Symptom Questionnaire (4DSQ). A questionnaire to measure distress, depression, anxiety, and somatization]. *Huisarts en Wetenschap*

**Warshaw MG, Keller MB, Stout RL** (1994). Reliability and validity of the longitudinal interval follow-up evaluation for assessing outcome of anxiety disorders. *Journal of Psychiatric Research* **28**, 531–545.

**Watson D, Weber K, Assenheimer JS, Clark L a, Strauss ME, McCormick R a** (1995). Testing a tripartite model: I. Evaluating the convergent and discriminant validity of anxiety and depression symptom scales. *Journal of abnormal psychology* **104**, 3–14.

**WHO Collaborating Centre for Drug Statistics Methodology** (n.d.). *ATC/DDD Index 2019*

**Wiersma JE, Hovens JGFM, Van Oppen P, Giltay EJ, Van Schaik DJF, Beekman ATF, Penninx BWJH** (2009). The importance of childhood trauma and childhood life events for chronicity of depression in adults. *Journal of Clinical Psychiatry*

**World Health Organisation** (1989). *Measuring obesity : classification and description of anthropometric data.* World Health Organization Regional Office for Europe: [Copenhagen].

**World Health Organization** (1998). *World Health Organization, Composite International Diagnostic Interview (CIDI), core version 2.1.* Geneva.
